# Creating flat-top X-ray beams by applying surface profiles of alternating curvature to deformable piezo bimorph mirrors

**DOI:** 10.1107/S1600577516013308

**Published:** 2016-10-12

**Authors:** John P. Sutter, Simon G. Alcock, Yogesh Kashyap, Ioana Nistea, Hongchang Wang, Kawal Sawhney

**Affiliations:** a Diamond Light Source Ltd, Harwell Science and Innovation Campus, Chilton, Didcot, Oxfordshire OX11 0DE, UK

**Keywords:** X-ray, bimorph, mirror, beam shaping, re-entrant

## Abstract

Beam shaping is becoming increasingly important for synchrotron X-ray applications. Although routine for visible light lasers, this is challenging for X-rays due to the limited source coherence and extreme optical tolerances required for the shaping mirrors. In deliberate defocusing, even surface errors <5 nm r.m.s. introduce damagingly large striations into the reflected beam. To counteract such problems, surface modifications with alternating concave and convex curvature on equal segments were polished onto the surface of non-active mirrors of fixed curvature. Such optics are useful for providing a fixed size of X-ray beam, but do not provide the adaptability required by many experiments. In contrast, deformable piezo bimorph mirrors permit a continuous range of X-ray beam sizes and shapes. A new theory is developed for applying non-periodic modifications of alternating curvature to optical surfaces. The position and length of the segments may be freely chosen. For the first time, surface modifications of alternating curvature are applied to bimorph mirrors to generate non-Gaussian X-ray beam profiles of specified width. The new theory’s freedom is exploited to choose the segments to match the polishing errors of medium wavelength (>10 mm) and the piezos’ influence on the mirror’s figure. Five- and seven-segment modifications of alternating curvature are calculated and verified by visible light and X-ray metrology. The latter yields beam profiles with less striation than those made by defocusing. Remaining beam striations are explained by applying geometrical optics to the deviations from the ideal surface modifications of alternating curvature.

## Abstract 2

A piezo bimorph mirror is deformed into three distinct re-entrant surface modifications as well as being simply defocused. A re-entrant modification with seven segments (the maximum possible for this mirror) produces an expanded beam with less striation than a simply defocused beam.

## 1. Introduction   

### 1.1. X-ray beam shaping: motives, requirements and solutions   

The ability to shape the transverse profile of photon beams, long since achieved in laser physics, is now being intensively pursued for X-rays generated at synchrotron light sources, including Diamond Light Source (Diamond), the UK’s national facility. Especially desirable are optics which can convert Gaussian X-ray beams produced by the various synchrotron sources (bending magnets, undulators or wigglers) into beams with a constant intensity ‘top-hat’ profile of selectable size from <1 to 100 µm. Synchrotron experiments often require a selectable width of X-ray beam to match to the sample size, or to achieve a required spatial resolution. For example, Diamond’s macromolecular crystallography beamlines I02, I03 and I04 and its small molecule single-crystal diffraction beamline I19 have all been equipped with the capability to control the beam size at the sample. All of these beamline teams consider a flat-top profile as ideal. Beam shaping is also important for the free-electron laser community, as demonstrated by the use of a deformable mirror for this purpose at the TIMEX beamline of FERMI@Elettra (Svetina *et al.*, 2011 ▸). Because it is more difficult to manufacture aspheric lenses for X-rays than for visible light, synchrotron beamline teams desiring top-hat X-ray beams have generally used mirrors. Due to their very shallow grazing angle of incidence, synchrotron X-ray mirrors can be up to 1.5 m in length. The short wavelength of X-rays, and the requirement for very small focal spots (micrometers or even tens of nanometers in diameter), mean that optical performance is strongly influenced by surface errors. The quality of synchrotron X-ray mirrors continues to improve and manufacturers can now routinely produce 1 m-long optics where the surface profile deviates by <<5 nm r.m.s. (the current state-of-the-art is ∼0.1 nm r.m.s.) from the required ellipse or cylinder. Height deviations of <1 nm r.m.s. correspond to slope deviations of <0.1 µrad r.m.s. However, even such extremely small errors are still a limiting factor for beamline performance, and hence efforts continue to create ever better X-ray mirrors.

**Figure 1:**
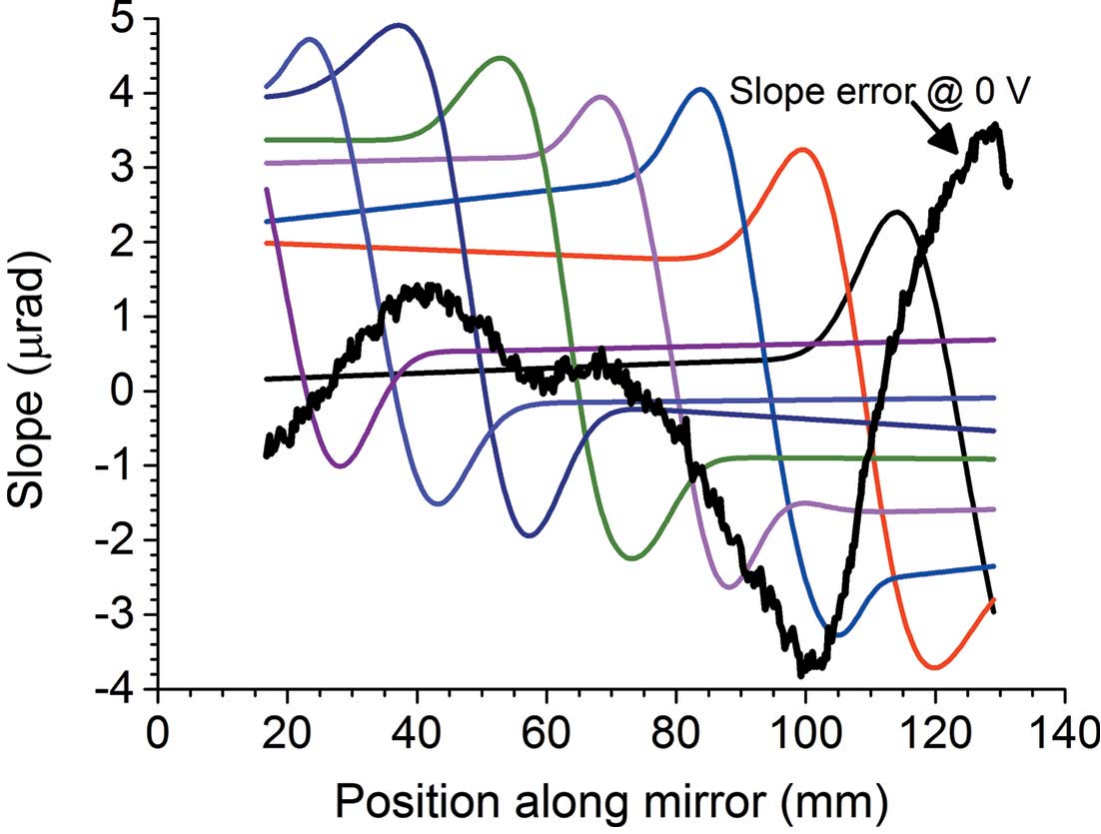
Piezo response functions (PRF), displayed as slope changes scaled linearly to a voltage change of 500 V for easy comparison, showing how each of the eight channels of a super-polished bimorph mirror affects the mirror’s surface, as measured by the Diamond-NOM profilometer (Alcock *et al.*, 2010 ▸). The thick black curve shows the mirror’s slope error (deviation from nominal ellipse) when all electrodes are at 0 V. The PRFs and the residual surface errors dictate the breakpoint locations of the re-entrant surface modifications.

Significant progress in X-ray beam shaping has been achieved through the development of deformable piezo bimorph mirrors (Susini *et al.*, 1995 ▸; Signorato *et al.*, 1998 ▸; Signorato & Ishikawa, 2001 ▸), which can be locally and globally bent by the application of controllable voltages to piezo ceramic rods or strips glued to optical substrates. Synchrotron bimorph mirrors typically have between 8 and 16 independent electrodes. The effect on the mirror’s figure of applying voltage to a given electrode is described by that electrode’s ‘piezo response function’ (PRF). Fig. 1 ▸ shows the PRFs for the 8-electrode bimorph mirror used in this study, and its polishing errors when zero volts are applied to all piezos. (Throughout this paper, ‘polishing errors’ will refer to surface errors of wavelength greater than approximately 10 mm left by the surface polishing process.) Unfortunately, when any active synchrotron X-ray mirror is purposefully bent such that the beam is not in focus, random polishing errors on the optical surface introduce striations into the defocused X-ray beam. The accurate measurement of such errors is important for understanding why this occurs. *In situ* (using X-rays) and *ex situ* (using visible light) metrology techniques have been developed at many synchrotrons since the late 1990s to characterize X-ray optics with sub-nanometer accuracy. Diamond itself utilizes *ex situ* interferometry and deflectometry (Diamond-NOM: Alcock *et al.*, 2010 ▸, 2013 ▸), in addition to *in situ* techniques such as pencil-beam scanning (Hignette *et al.*, 1997 ▸; Sutter *et al.*, 2011 ▸), grating interferometry (Wang *et al.*, 2014 ▸) and X-ray speckle tracking (Berujon *et al.*, 2014 ▸; Wang, Kashyap *et al.*, 2015 ▸; Wang, Sutter *et al.*, 2015 ▸) at the Versatile Test Beamline B16 (Sawhney *et al.*, 2010 ▸).

### 1.2. Shaping of beams by shaping of mirrors   

Spiga *et al.* (2013 ▸) were the first to determine how an X-ray mirror should be deformed to convert an X-ray beam from one profile to another. They calculated deformations where the second derivative of height (*i.e.* curvature) was positive along the mirror’s length. Such deformations are frequently applied to bimorph mirrors at Diamond (Sutter *et al.*, 2014 ▸). However, they also highlighted that an infinite number of deformations will also modify the beam profile in a similar way. These other deformations, which consist of concave and convex regions, will be called ‘re-entrant’ in the following treatment.

Nicolas & García (2013 ▸) showed that if a uniformily concave or convex surface deformation is applied to a mirror, the reflected X-ray beam’s striations depend on the unique spatial properties of the optical polishing errors, and cannot be adequately quantified using a single parameter. Even for state-of-the-art X-ray mirrors, beam striations are still a limiting factor, which necessitates novel surface modifications. The use of re-entrant surface modifications was first proposed by Laundy *et al.* (2015 ▸). These have continuous height and slope along the mirror, but their curvature switches sign discontinuously at the endpoints of segments of equal length. Because the curvature of these modifications does not pass through zero, these authors avoid spikes at the edges of the reflected beam profiles that are predicted by Spiga *et al.* (2013 ▸) when sinusoidal surface modifications are used. The exact solution is given for broadening the beam at the focus to a specified size in the case of uniform illumination along the mirror’s length. Ray traces by Laundy *et al.* (2015 ▸) indicate that polishing errors added to re-entrant surface modifications introduce less striation into the beam at the focus than the same errors added to a surface modification of uniform curvature. The X-ray beam also grows more uniform as the number of segments increases, which can be explained by the overlaying of striations from different segments. If the errors have no correlated texture, the striations produced by different segments will average out. Because a real deformable mirror can never change its curvature discontinuously at any point, some spikes will inevitably be introduced to the beam profile by the regions of the mirror where the curvature of the modification passes through zero. Examples of this will be modeled theoretically in §4.4 and compared with the experimental beam profiles.

A fixed-shape mirror prototype was tested with X-rays by Laundy *et al.* (2016 ▸). Three lanes were polished into this mirror, two with re-entrant surfaces of four equal segments, optimized for focusing the beam to a spot size of <1 µm, 2 µm or 10 µm. The three-lane mirror permits rapid switching of beam size, but only to three discrete sizes of a given shape. By contrast, adjustment of the beam shape and size at the focus through a continuous range can be achieved using the fine control of local curvatures provided by deformable piezo bimorph mirrors. For this study, an uncoated 150 mm-long deformable piezo bimorph mirror with a silica substrate and eight electrodes was used. The central 115 mm of the substrate was ‘super-polished’ by elastic emission machining (EEM) (Yamauchi *et al.*, 2002 ▸) by JTEC (Japan) to an elliptical cylinder of source–mirror distance 41.5 m, mirror–image distance 0.4 m, and grazing incidence angle 3 mrad. Thales-SESO (France) assembled the substrate into a bimorph mirror. The mirror has been measured extensively over >5 years (Sawhney *et al.*, 2013 ▸) showing that its surface profile and its PRFs have remained stable. X-ray measurements were performed at the Diamond beamline B16, which uses a bending magnet to generate X-ray beams of which the intensity distributions are independent of horizontal position. Because the mirror deflects and focuses vertically, only the reflected beam’s vertical profile is significantly affected. Furthermore, the mirror’s aperture, (150 mm)(3 mrad) = 450 µm, is much smaller than the vertical width of the bending magnet beam. Therefore, the mirror is uniformly illuminated along its length. The design of bimorph mirrors imposes two restrictions on possible surface modifications. Firstly, the piezos are least able to correct polishing errors at the junctions between adjacent piezos. Secondly, the voltage difference between adjacent electrodes cannot exceed 500 V without risk of damage to the mirror. This makes it advantageous to purposely choose the breakpoints between re-entrant segments to lie at the junctions between adjacent electrodes. However, not every junction needs to be a breakpoint. By placing the breakpoints only at certain junctions, one can make the re-entrant surface modification approximately match the mirror’s figure errors at 0 V, thus minimizing the voltages required to deform the mirror (see Fig. 1 ▸). This leads to an innovation: re-entrant surface modification segments that are given unequal lengths by controlling groups of electrodes together. In the following, five- and seven-segment re-entrant surface modifications will be investigated for X-ray beam shaping. No re-entrant modifications with as many segments have ever been tested. Furthermore, the new theory presented here can generate re-entrant surface modifications on any arbitrary set of segments without alteration of the reflected beam’s size or shape, thus extending its validity beyond the bimorph mirrors presented here as an example.

### 1.3. Modeling and evaluating expanded beam profiles using measured mirror surface profiles   

Simulations of the profiles of X-ray beams reflected from real mirrors are less advanced than those of lasers because of the synchrotron X-rays’ limited coherence. While lasers may have temporal coherence lengths from millimeters to tens of meters, synchrotron X-rays generally have temporal coherence lengths of a few micrometers after passage through a double-crystal monochromator (bandpass Δλ/λ ≃ 10^−4^). X-rays produced by synchrotrons also do not approach perfect spatial coherence as nearly as laser beams do, although X-ray beams from undulators can have quite high spatial coherence in the vertical direction at the relatively low photon energies of this paper. Nevertheless, one should consider that undesirable wavelength-dependent diffraction effects can appear when the surface of a focusing mirror is modulated. These arise from the resulting modulation in the path length of the X-rays reflected from the mirror. Physical optics must therefore be used in principle, but Laundy *et al.* (2015 ▸) made some simple calculations for the case of a periodic modification of wavelength Λ, which behaves like a diffraction grating. If θ is the grazing incidence angle of the X-rays on the mirror, the phase of the reflected wavefront is modulated with a period Λsinθ. X-rays with a wavelength λ will then form diffraction peaks in the focal plane, which is at a distance *q* from the mirror. The spacing of the diffraction peaks is *q*λ/(Λsinθ). If this interval is much less than the focal spot size Σ of the unmodified surface, the diffraction peaks overlap and diffraction effects in the focal plane are smeared out. This will occur if Λ >> *q*λ/(Σsinθ). Geometrical optics can then be used to model the beam profile at the focus. In the examples of this paper, X-rays of 8000 eV (λ = 1.55 Å) and 9200 eV (λ = 1.35 Å) were reflected from the mirror. The focal distance *q* is approximately 0.4 m, the grazing incidence angle θ is approximately 3 mrad, and the focal spot size Σ produced by the unmodified surface is approximately 1 µm. For the worst case of 8000 eV X-rays, one derives the condition Λ >> 20.67 mm for geometrical optics to be valid in calculating the beam profile at the focus. 9200 eV X-rays yield the corresponding condition Λ >> 18 mm. The surface modifications of this paper are not periodic, but they will have wavelengths Λ no shorter than 30 mm. Diffraction effects are therefore not expected to be strong.

Geometrical optics have been applied by Spiga *et al.* (2013 ▸) and Nicolas & García (2013 ▸) to explain many features of X-ray beams reflected from imperfect mirrors. Indeed, an elementary ray-tracing model applied by Sutter *et al.* (2014 ▸) to the measured figure of a bimorph mirror at Diamond yielded theoretical reflected beam profiles in good agreement with X-ray knife-edge measurements at the undulator beamline I02. Measured profiles of the reflected beam will be compared with ray tracing calculations using *SHADOW* (Sanchez del Rio *et al.*, 2011 ▸). Although perfect matches are not obtained because of slight variations in the detector’s position and the X-rays’ incidence angle, ray tracing explains many prominent features of the measured profiles. Moreover, although ideal top-hat profiles have not been achieved, geometrical optics provide important clues for improving X-ray profiles.

A future verification of the results of geometrical optics can be directly obtained from the more general theory of physical optics. The wave behavior of X-rays has been taken into account by Laundy *et al.* (2016 ▸), who applied the Fresnel–Kirchhoff equation (Born & Wolf, 1999 ▸) to a simple physical model. A more comprehensive treatment using physical optics to treat mirror imperfections of arbitrary scale was reported by Spiga & Raimondi (2014 ▸) and by Raimondi & Spiga (2015 ▸). They used their own IDL code called *WISE*. Other software packages for physical optics calculations on synchrotron radiation, such as *PHASE* (Bahrdt, 1997 ▸), *FOCUS* (Bowler & Higgins, 2009 ▸), *WAVE* (Scheer, 2008 ▸) and *SRW* (Chubar & Elleaume, 1998 ▸), also exist. We note, however, that modeling the propagation of partially coherent X-rays through imperfect optics is a subject of active research. Software for X-ray physical optics continues to improve, and Diamond has assisted in the expansion of the *SRW* program.

Because X-ray beam shaping is in its infancy, no universally accepted quantitative metric yet exists for judging how accurately a measured beam profile approaches the desired shape. This is not an unprecedented situation in optics. For example, Shealy & Hoffnagle (2005 ▸) mention the lack of a quantitative figure of merit for the propagation behavior of laser beams. In the following, the desired shape is a top-hat, which in laser literature has been approximated by a variety of functions. Here, the measured X-ray beam profiles will be fitted to super-Gaussians. Such fits produce two figures of merit: the degree of flatness and striation. Although imperfect, they will allow more objective comparisons of profiles than subjective judgments made ‘by eye’.

### 1.4. Outline   

Re-entrant modifications with a variable number of segments of arbitrary length will be calculated under the assumption that the incident X-ray intensity is uniform over the whole mirror. This assumption is justified by the mirror’s small aperture. Three such modifications, each determined for a 4 µm-wide beam at focus, will then be applied to the bimorph mirror described above, and an *ex situ* measurement will show how nearly the bimorph mirror actually matches each. Special attention will be paid to the region around each breakpoint, since, as Spiga *et al.* (2013 ▸) showed, a spike will be generated in the beam profile if the curvature passes through zero. Any other regions of zero curvature must also be taken into account. X-ray speckle tracking will confirm that the re-entrant surface applied *in situ* approximately matches the one applied *ex situ*, though small discrepancies due to slightly varying temperature, mounting forces and angles of incidence are expected. For two of these modifications, the beam profile at the focus will be measured, and the quality of each will be estimated by fitting it to a super-Gaussian,
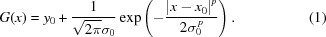
Finally, imperfections in all the measured beam profiles at the focus will be compared with geometrical optics simulations using measured deviations of the bimorph mirror’s actual profile compared with the desired ideal re-entrant figure, thus showing how future mirrors could be better manufactured. Some additional considerations are discussed by Sutter *et al.* (2016 ▸).

## 2. Principles   

### 2.1. Re-entrant surface modifications on segments of unequal length   

**Figure 2:**
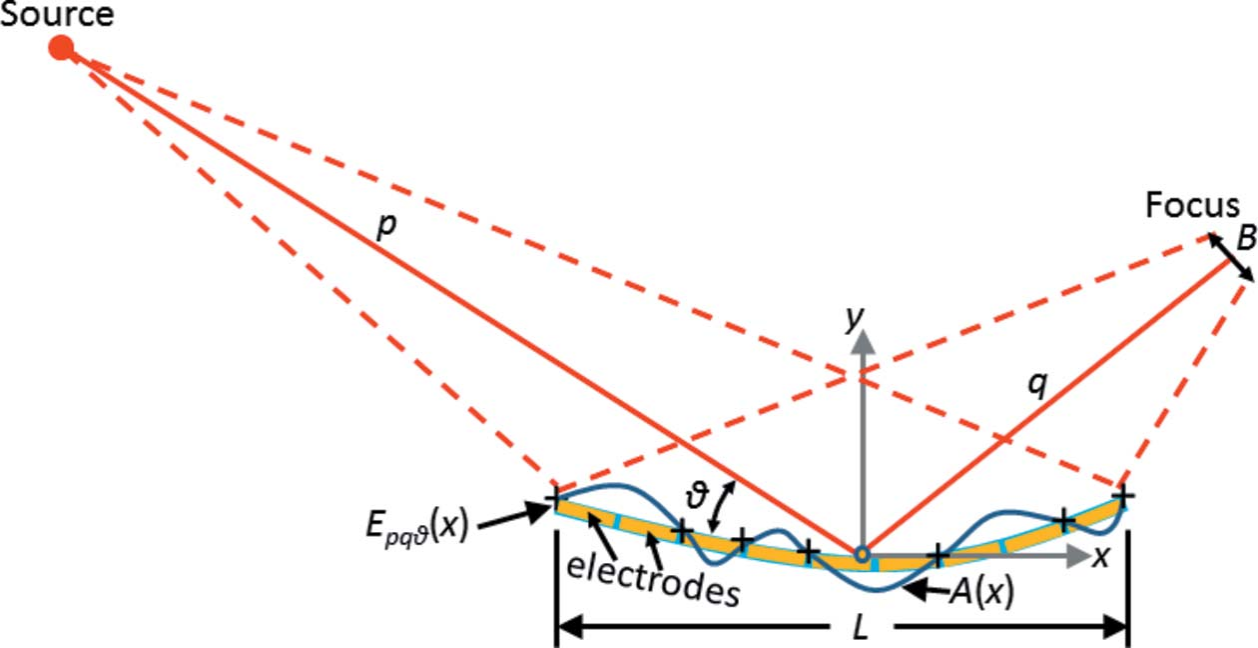
Schematic showing how the variables defined in §2.1 are associated with the application of a re-entrant surface modification to a bimorph mirror. This example has nine electrodes that are used to apply a re-entrant modification of six segments. The breakpoints of the surface modification *A*(*x*) are indicated by the + symbols. They are located at the points of maximum and minimum values of *A*′(*x*). See text for details.

We develop a new theory, expanding on previous work by Laundy *et al.* (2015 ▸), to create re-entrant surface modifications with segments of unequal lengths. The novelty of this approach enables the breakpoints to be arbitrarily chosen. For our application, using bimorph mirrors, we chose the breakpoints to coincide with the location of polishing errors and regions where the bimorph’s piezos influence the optical surface. A schematic explaining the procedure of this section is displayed in Fig. 2 ▸. To expand the X-ray beam at the focus to a size *B*, consider a mirror of length *L* with *n* segments. Let *q* be the distance from the center of the mirror to the focus, and θ be the grazing angle of the incident beam at the mirror’s center. Let the abscissa *x* denote position along the length of the mirror with positive *x* toward the focus, and *x* = 0 be the center of the mirror. Define the breakpoints *x*_*j*_ where *j* = 0,…, *n* such that
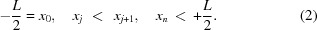

*x*_1_ to *x*_*n*–1_ are initially selected as the junctions between adjacent electrodes. The center of each electrode is defined as the midpoint between the maximum and minimum slope of its PRF, as illustrated in Fig. 1 ▸. Note, however, that the theory is flexible in that the breakpoints can be iteratively shifted to better fit the PRFs without any effect on the beam size. For each segment, define *S*_*i*_ where *i* = 1,…, *n* such that
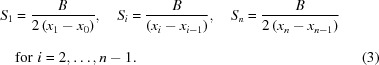
The figure of the mirror will be given by a function *y*(*x*):
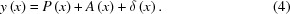

*P*(*x*) is an ideal pre-ground shape of the mirror. In this paper, it will be equal to *E*_*pq*θ_(*x*), the ellipse specified by the source–mirror distance *p*, the mirror–image distance *q*, and the grazing incidence angle θ at the center of the mirror. The formula for *E*_*pq*θ_(*x*) is given in equation (15) of Sutter *et al.* (2010 ▸), with the sign of the abscissa switched to account for the reversed direction of the *x*-axis:
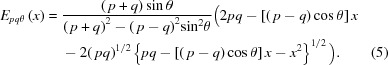

*A*(*x*) is a purposely induced change in the mirror’s figure from the simple function *P*(*x*). In this paper, *A*(*x*) will be the re-entrant surface modification. Finally, δ(*x*) is the surface error left behind by imperfect figuring. It will be assumed equal to zero for the following treatment.

In this model, the detector will be placed at the focus of the ideal ellipse and oriented at right angles to the central reflected ray. The X-ray source’s horizontal and vertical profiles are Gaussian with r.m.s. widths of 34.5 µm and 7.0 µm, respectively. With *p* = 41.5 m and *q* = 0.4 m as stated in §1.2, the demagnified r.m.s. vertical source size at the mirror’s focus is (7.0 µm)(*q*/*p*) = 0.067 µm, less than the step size of the knife-edge scans that will be reported in this article. Therefore the source size is not expected to affect the measured profiles significantly, and the source may be treated here as a point. Finally, because the mirror is very far from the source and because its aperture of approximately 450 µm is much smaller than the vertical width of the bending magnet beam (see §1.2), the illumination of the mirror will be assumed uniform over the mirror’s surface. Note, however, that the treatment of this section can easily be generalized to other spatial distributions of X-ray flux on the mirror, although the resulting differential equations may not necessarily have analytical solutions.

Because the ray deflection from X-ray mirrors is always small, to first order in *x* a ray reflected from the mirror at *x* reaches the detector at
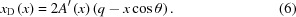
If the flux on the mirror from *x* to *x* + d*x* is *I*_mirr_(*x*)d*x*, and if the flux on the detector from *x*_D_ to *x*_D_ + d*x*_D_ is *I*_det_(*x*_D_)d*x*_D_, then these two fluxes can be set equal on any segment of the re-entrant surface modification, since there d*x*_D_/d*x* is either less than zero or greater than zero along the segment’s entire length excluding the breakpoints. Suppose one examines the *i*th segment, which extends along the range (*x*_*i*–1_, *x*_*i*_). In this range one would obtain a differential equation
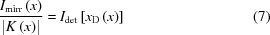
where
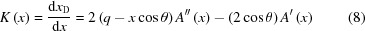
is determined by applying the chain rule to equation (6).

Since the illumination is uniform, *I*_mirr_(*x*) = 1 for −*L*/2 ≤ *x* ≤ +*L*/2. For a top-hat beam of width *B*, *K*(*x*) on the *i*th segment is set equal to (−1)^*i*+1^ *S*_*i*_, with *S*_*i*_ given by equation (3). By substituting this value of *K*(*x*) into equation (8), one obtains a differential equation of which the terms in *x* and *A*′(*x*) can be separated and integrated. The resulting slope 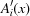 on the *i*th segment is thus
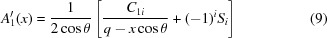
and a second integration over *x* yields the height *A*_*i*_(*x*) of the *i*th segment:
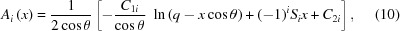
where the 2*n* constants of integration *C*_1*i*_ and *C*_2*i*_ are determined by the boundary conditions
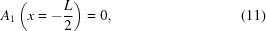


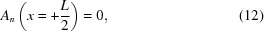


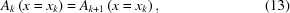


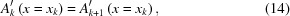
and where *k* = 1,…, *n* − 1. Thus there are 2*n* equations to determine the 2*n* constants of integration, calculated using *MATLAB* (MathWorks, 2004 ▸). Note that equation (13) guarantees continuity of the height across segments, while equation (14) guarantees continuity of the slope. However, the curvature *A*′′(*x*) is allowed to be discontinuous at the breakpoints.

Note that equation (10) can be expanded in a Taylor series about *x*_*i*_. Since *L*/2 << *q* in this paper, one need expand only to second order, since quadratics are the lowest-order polynomials from which re-entrant modifications of continuous height and slope can be constructed. The result is as follows:
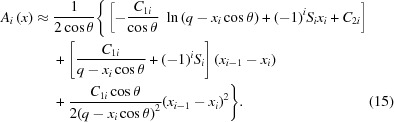
The re-entrant modification thus reduces to a series of parabolic arcs connected at the breakpoints.

It should be stressed that, even though this calculation has been developed with bimorph mirrors in mind, it is not limited to bimorph mirrors because the breakpoints *x*_*j*_ can be arbitrarily chosen. Thus, for example, one could imagine a mirror of fixed shape like the prototype tested by Laundy *et al.* (2016 ▸), but with its breakpoints distributed in any arbitrary configuration. The equations of this section would still be valid. It is only the design of a bimorph that forces the breakpoints to be set at the junctions, as will be done in §2.2.

### 2.2. Re-entrant surface modifications applied to a bimorph mirror   

Re-entrant surface modifications applied to the EEM bimorph mirror were chosen by examination of the mirror’s surface polishing errors and the piezo response functions as measured using the Diamond-NOM (Alcock *et al.*, 2010 ▸). The PRFs were determined according to the procedure described by Hignette *et al.* (1997 ▸) and Sutter *et al.* (2011 ▸), beginning with all voltages set to 0 V and using a +200 V increment. Voltage optimization was performed by the linear algebra method of least-squares minimization.

**Figure 3:**
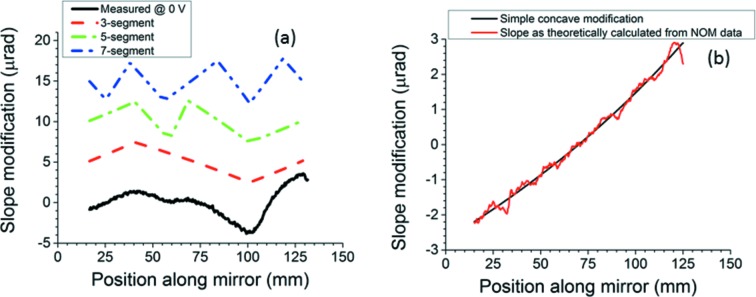
(*a*) Bottom to top: mirror slope error at 0 V, then theoretical three-, five- and seven-segment surface modifications to try to mimic the slope error at 0 V. Each plot has been shifted upward by 5 µrad from the one before for easy comparison. (*b*) Theoretical simple concave surface modification with expected best approximation achieved by mirror as calculated from its slope error at 0 V and its measured PRFs. The calculated voltage settings first remove the polishing errors and then add the desired modification. Intended beam size is 4 µm for all cases.

On a bimorph mirror, it is not useful to place a breakpoint within the area influenced by any particular electrode, because the figure within that area can be corrected simply by applying a suitable voltage, and also because the curvature within that area will be uniform to a good approximation. The only useful sites for breakpoints are at the electrode junctions, for only there can the bimorph mirror approach the ideal discontinuous switch in the curvature’s sign. This requirement sets a finite limit on the number of re-entrant surface modifications with a given number of segments that can be applied to a bimorph. Once the possible breakpoints have been determined from the PRFs in Fig. 1 ▸ as the midpoints between the centers of adjacent electrodes, the first breakpoints must be placed at those junctions where the derivative of the slope error at 0 V most abruptly switches sign. For the mirror of this experiment, these will be at the junctions between electrodes 2 and 3 and electrodes 6 and 7. If one proceeds no further, one has already generated a three-segment modification. For more segments, additional breakpoints can be placed so as to follow the figure error when all electrodes are at 0 V, because the mirror can be deformed to that shape with the least voltage change. A somewhat subjective judgment of the best re-entrant modification has been made, and therefore the possibility that better modifications exist is still open, but selected surface modifications are displayed in Fig. 3(*a*) ▸, along with the measured slope error at 0 V after subtraction of the best-fit line (curvature). For comparison, Fig. 3(*b*) ▸ shows the more common type of surface modification for beam enlargement: a simple concave defocus. All modifications are designed to expand the beam size to 4 µm at the mirror’s focus.

## 3. Measurements of surface modifications with the Diamond-NOM   

**Figure 4:**
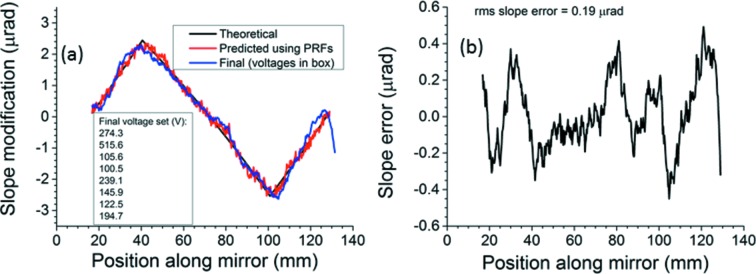
(*a*) Three-segment surface modification: theoretical, predicted and measured using the Diamond-NOM. (*b*) Slope error (deviation of measured surface modification from theoretical one).

The next tasks are to measure the accuracy with which the bimorph mirror can reproduce each requested re-entrant surface modification, and to understand the limiting factors. The simplest three-segment re-entrant modification is shown in Fig. 4 ▸. The list of voltages in Fig. 4(*a*) ▸ confirms that all voltage differences between adjacent electrodes remain within the 500 V limit. The breakpoints, including the ends, are at 16.75, 40.5, 100.75 and 129 mm.

The bimorph mirror could not always be deformed exactly to the re-entrant modification given the initially selected breakpoints, but it could more nearly approach a different re-entrant modification yielding the same beam size but with slightly shifted breakpoints. In these cases, the closest achieved modification was measured, then compared with a new theoretical re-entrant modification of which the breakpoints are displaced to the maxima and minima of the measured slope.

**Figure 5:**
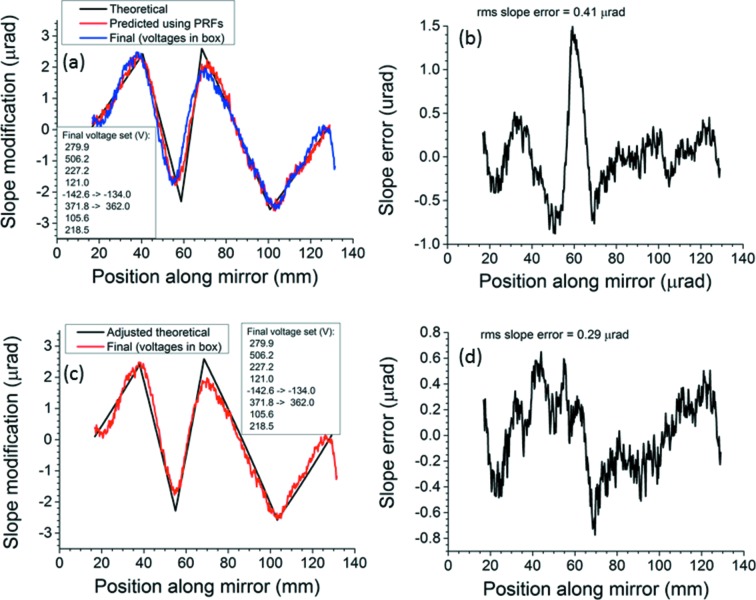
Five-segment surface modification. (*a*) Initial breakpoints: theoretical, predicted and as measured. (*b*) Difference between theoretical and measured for the initial choice of breakpoints. (*c*) Breakpoints adjusted to improve the match between theoretical and measured. (*d*) Difference between theoretical and measured for the final choice of breakpoints. Arrows in the voltage lists in (*a*) and (*c*) show where ideal voltages had to be compromised to ensure voltage differentials remain below the 500 V limit.

This process is demonstrated in Fig. 5 ▸ for the five-segment modification. Fig. 5(*a*) ▸ compares the initial theoretical surface modification with the predicted and the measured ones. Fig. 5(*b*) ▸ shows a large peak in the slope error at 60 mm along the mirror. Therefore the breakpoints were shifted onto extrema of the final achieved modification. Figs. 5(*c*) and 5(*d*) ▸ show that the EEM bimorph matched the new modification much better. The voltage on electrode 5 was raised by 8.6 V and the voltage on electrode 6 was lowered by 9.8 V in order to keep the voltage differential below the 500 V limit. This adjustment slightly shifts the maxima and minima of the achieved slope modification, but still keeps the voltages close to the theoretical optimum. The final breakpoints, including the ends, lie at 16.75, 38, 55, 68.5, 103.25 and 129 mm.

**Figure 6:**
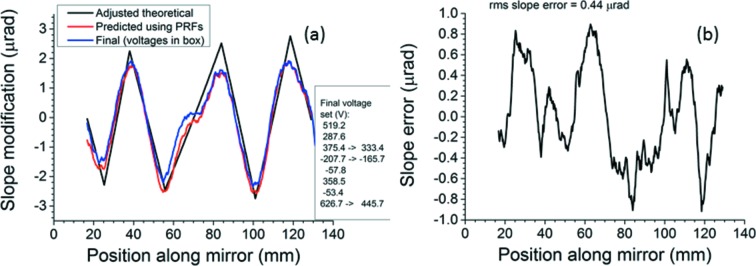
Seven-segment surface modification. (*a*) Adjusted theoretical, predicted and measured. (*b*) Slope error (final minus adjusted theoretical). Arrows in the voltage list in (*a*) show where the voltages had to be shifted away from the theoretically optimal values in order to keep the voltage differentials below the 500 V limit.

The seven-segment modification was the most difficult to achieve because sharp changes in curvature are necessary between every pair of adjacent electrodes except 4 and 5, which are joined together. Finding the optimal seven-segment modification required the same adjustment of the breakpoints’ positions to match the measured data as was done for the five-segment modification. Fig. 6(*a*) ▸ compares the final seven-segment modification with the adjusted theoretical one and the one predicted using the PRFs. The slope error of the final measured modification with respect to the adjusted theoretical is shown in Fig. 6(*b*) ▸. To keep within the maximum voltage differential of 500 V, the voltage on electrode 3 was lowered by 42 V and the voltage on electrode 4 was raised by the same amount. Also, the voltage on electrode 8 was lowered from 626.7 V to 445.7 V, although this alteration at the end of the mirror is less significant than one near the center. As before, these adjustments will shift the slope maxima and minima upward or downward. The final breakpoints, including the ends, lie at 16.75, 25.25, 38, 55.75, 84, 101, 118.5 and 129 mm.

Agreement of the final measured surface modifications with the adjusted theoretical ones is good throughout. The slope errors are 0.19 µrad r.m.s. for three segments, 0.29 µrad for five segments and 0.44 µrad for seven segments. Further reduction of these slope errors by additional iterations appears unlikely as a good match to the theoretical modification has already been produced in all three cases. As one might expect, a truly discontinuous change in the sign of the curvature at the breakpoints is not achieved; some rounding of the slope extrema is always present in the measured modifications. Finally, the measured seven-segment modification is flattened at the junction between electrodes 4 and 5. A similar, though less pronounced, flattening is visible at the center of the mirror when the three-segment modification is applied. This is an ‘imprint’ effect caused by the finite width of electrodes, as previously observed by Alcock *et al.* (2013 ▸) and discussed more fully by Sutter *et al.* (2014 ▸).

## 4. Measurements of X-ray beam striation introduced by re-entrant surface modification   

### 4.1. Experimental procedures   

**Figure 7:**
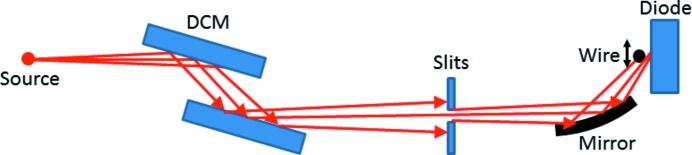
Schematic of the experimental setup used to measure the reflected X-ray beam profiles in the vicinity of the focus of the bimorph mirror at Diamond’s Versatile Test Beamline B16 (Sawhney *et al.*, 2010 ▸). (The figure is not to scale.) The incidence angle of the beam on the mirror is shown here for clarity as much larger than its true value of 3 mrad.

Following *ex situ* profilometry using the Diamond-NOM, five- and seven-segment modifications were examined with X-rays at the Diamond Light Source Versatile Test Beamline B16 (Sawhney *et al.*, 2010 ▸). The experimental setup is shown schematically in Fig. 7 ▸. X-rays with a photon energy of 9200 eV were selected by a Si (1 1 1) double-crystal monochromator (DCM). A series of slits downstream of the DCM selected a 0.03 mm (horizontal) × 0.3 mm (vertical) section of the X-ray beam produced by the bending magnet source. The EEM bimorph mirror was oriented for upward deflection inside a closed chamber that was continually flushed with N_2_ gas at a rate of 1 L min^−1^ to prevent the generation of reactive hydrocarbon atoms which can contaminate the optical surface. Kapton windows allowed the X-rays to enter and exit the sealed chamber. At a grazing incidence angle of 3 mrad, the incident beam illuminated the entire mirror’s active area.

For each surface modification, striations in the X-ray beam were measured by knife-edge scans using a horizontal gold wire. The 200 µm-diameter wire was scanned through the reflected beam, at distances in 0.2 mm intervals from 384 to 388 mm downstream from the center of the mirror. Previous measurements had shown that the focus of the mirror was within this range. In this way, the dependence of the beam size and striations on detector distance from the mirror in the neighborhood of the focus is clearly observed. The wire was vertically translated in 100 nm steps by a piezo stage. The narrow horizontal width of the slits upstream of the mirror ensures that the striations are not blurred by sagittal slope errors on the mirror, or by accidental inclination of the gold wire. X-ray intensity behind the gold wire was measured by a PIPS detector with a current amplifier.

Results are compared with knife-edge scans of the reflected beam striations produced when the surface modification was a simple variation of curvature. These scans had been collected with a step size of 0.2 µm using X-rays of 8000 eV, but all other experimental conditions were the same except for possible slight variations in temperature and mounting forces. Because striations of the experimental plots extend over widths several times greater than 0.2 µm, the difference in scan step size is not expected to influence the results substantially. As will be shown later, the main striations in the wire scans for the re-entrant modifications can be explained by geometrical theory and therefore should be very similar for X-rays of 8000 eV or 9200 eV. In addition, although the earlier wire scans were taken two and a half years before the current ones, repeated Diamond-NOM examinations of the mirror within that time showed no change in the mirror’s figure or electrode responses.

Because experimental beam profiles are determined by calculating the derivatives of the knife-edge scans numerically using finite differences, one may ask how susceptible they are to measurement noise. Each knife-edge scan for the simple mirror curvature changes was repeated three times. Comparison of their derivatives showed they were reproducible; therefore, the three scans were averaged. This averaging was not performed on the knife-edge scans when re-entrant modifications were applied to the mirror. However, the fact that in consecutive data sets the striations and the peak shape change only gradually clearly shows that the measured structure is real and not random experimental noise. Additional data sets (not shown here) taken with other mirror surface modifications corroborate this. The reader should note that no smoothing of the numerical derivative was performed in any of the knife-edge scans in this paper.

Previous attempts to measure the mirror surface using X-ray pencil-beam scans were limited by the insensitivity to surface slope at the short focal length of 0.4 m. The X-ray speckle scanning technique (Berujon *et al.*, 2014 ▸; Wang, Kashyap *et al.*, 2015 ▸) was therefore used. It is not expected that the Diamond-NOM and X-ray speckle tracking will produce exact agreement, partly because the latter technique is still being perfected, and partly because the mirror is not under precisely the same experimental conditions. However, the two datasets confirm that the application of the voltages determined by the Diamond-NOM produced sufficiently similar mirror surface modifications at B16. A diffuser consisting of P3000 sandpaper was placed upstream of the EEM bimorph mirror to generate a speckle pattern in the reflected beam. The position of the sandpaper relative to the mirror was not critical, but was placed in close proximity to the mirror to minimize wavefront spread caused by the incident beam’s divergence. Then, with the horizontal width of the upstream slits opened to 0.5 mm, the sandpaper was translated vertically in 0.25 µm steps with a piezo stage. At each of the 80 steps, an image of the speckle pattern in the reflected beam was measured by a Photonic Science PSL-FDS X-ray camera (pixel size: 6.45 µm) placed 3.6 m downstream from the mirror. Motion of the speckles during each scan was tracked to determine the deviation of the mirror surface slope from that required for exact focusing.

### 4.2. Figures of merit for experimental beam profiles: the super-Gaussian function   

The super-Gaussian function *G*(*x*) in equation (1) is frequently used to describe higher-order laser beam modes that approach a rectangular cross-section. For *p* = 2, *G*(*x*) is an ordinary Gaussian function. Small values of *p* yield a function with a sharp peak and long tails, while large values of *p* produce a flat top. The full width at half-maximum (FWHM) of *G*(*x*) is 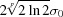. In the following discussion, the experimental X-ray beam profiles are fitted to super-Gaussians with free parameters *x*_0_, *y*_0_, σ_0_ and *p*. Three figures of merit emerge:

(i) σ_0_, which determines the width of the profile;

(ii) *p*, which determines the flatness of the profile’s top;

(iii) *R* ^2^, the ‘adjusted *R* squared’ value. Values from 0 to 1 indicate how well variance in the experimental data is explained by the model (0 not at all, 1 entirely).

Given a general set of measured data points (*X*_*i*_, *Y*_*i*_), *i* = 1,…, *N* and a fitting function *Y* = *f*(*X*,θ), where (θ_1_,…, θ_*k*_) is the set of fitting parameters, the formula for *R* ^2^ is
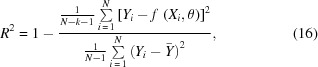
where 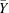 is the mean of the data points *Y*_*i*_. *R* ^2^ thus includes the mean square deviation from the fit, but is a more reliable measure of the goodness of fit because it is adjusted for the number of data points, the number of fitting parameters and the magnitude of the data points.

The ideal experimental beam profile would be a flat top of some desired width without striations. A measured profile approaches this most closely if its best-fit super-Gaussian has the correct width, a large *p*, and *R* ^2^ approaching 1. To a good approximation, if the tails are neglected, *R* ^2^ will be closely related to the amount of striation in the experimental profile: the closer it is to 1, the less striation is present. Fits were performed using *Origin Pro 9.0.0* (OriginLab Corporation, 2012 ▸).

### 4.3. Surface modification data and X-ray beam profiles at focus   

**Figure 8:**
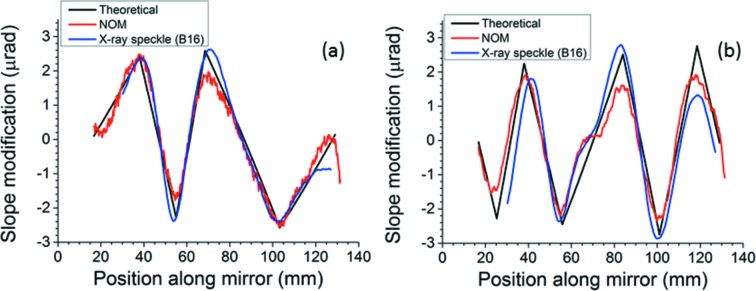
(*a*) Five-segment and (*b*) seven-segment surface modifications: comparisons of theoretical with measurements by Diamond-NOM and by X-ray speckle tracking at B16. The data measured on B16 are represented by the smooth curve with the rounded extrema.

Figs. 8(*a*) and 8(*b*) ▸ compare, respectively, the five- and seven-segment surface modifications with measurements on the Diamond-NOM and X-ray speckle tracking at B16. Although in good agreement, they deviate most from the theoretical curves, and from each other, close to the breakpoints where the measured curvature is significantly rounded. The imprint effect observed in the central segment of the seven-segment modification on the Diamond-NOM is also visible, though less pronounced, in the X-ray speckle tracking data.

**Figure 9:**
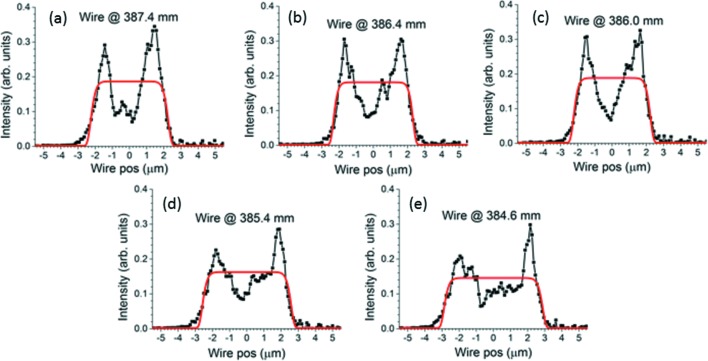
Five-segment surface modification: survey of beam width and striation near focus. Derivatives of knife-edge scans taken at labeled distances downstream from the center of the mirror chamber. Black curves with dots are measured profiles. Thick solid red curves are best-fit super-Gaussians.

To investigate the beam size and striations, the derivatives of the knife-edge scans need to be plotted and examined. Representative subsets of the results for the five- and seven-segment modifications are displayed in Figs. 9 ▸ and 10 ▸, along with their best-fit super-Gaussians. The structure of the reflected beam at the focus when the five-segment modification is applied is dominated by intense spikes at both edges. Such ‘hot edges’ are typical of continuous (rather than piecewise continuous) slope modifications, such as sinusoids. They arise from the regions of minimum and maximum slope where the position of the reflected ray on the detector varies minimally with the position of the corresponding incident ray along the mirror. This indicates that the rounding of the curvature close to the breakpoints of the five-segment curve strongly affects the beam profile. However, aside from this, very little striation is evident in the X-ray beam near focus. The structure of the beam profile remains very stable when the knife-edge’s distance from the mirror is varied. The hot edges are much less severe in the reflected beam produced by the seven-segment modification. This is partly because the measured extrema in Fig. 8(*b*) ▸ are not all at the same value of the slope, but also because of the sharper change in curvature at the breakpoints.

**Figure 10:**
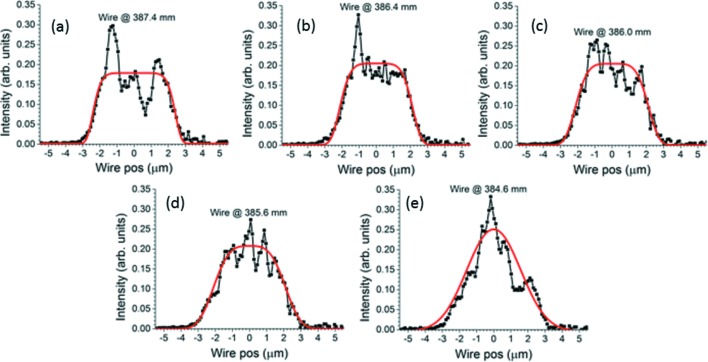
Seven-segment surface modification: survey of beam width and striation near focus. Derivatives of knife-edge scans taken at labeled distances downstream from the center of the mirror chamber. Black curves with dots are measured profiles. Thick solid red curves are best-fit super-Gaussians.

**Figure 11:**
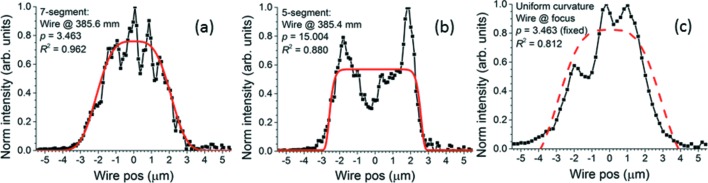
(*a*) Seven-segment for 4 µm beam, (*b*) five-segment for 4 µm beam, (*c*) simple concave for 5 µm beam: comparison of fits to super-Gaussians of representative knife-edge scans. Maximum experimental intensity normalized to unity for easy comparison of plots. Wire distance from center of mirror chamber, *p* and *R* ^2^ are in the labels. Black curves with dots are measured profiles. Thick solid red curves are best-fit super-Gaussians. Thick dashed red curve in (*c*) is best-fit super-Gaussian for which *p* is fixed at the value in (*a*). This is done for comparison of flatness and striation with (*a*). Note the decreasing goodness of fit *R* ^2^ as the number of segments decreases.

**Figure 12:**
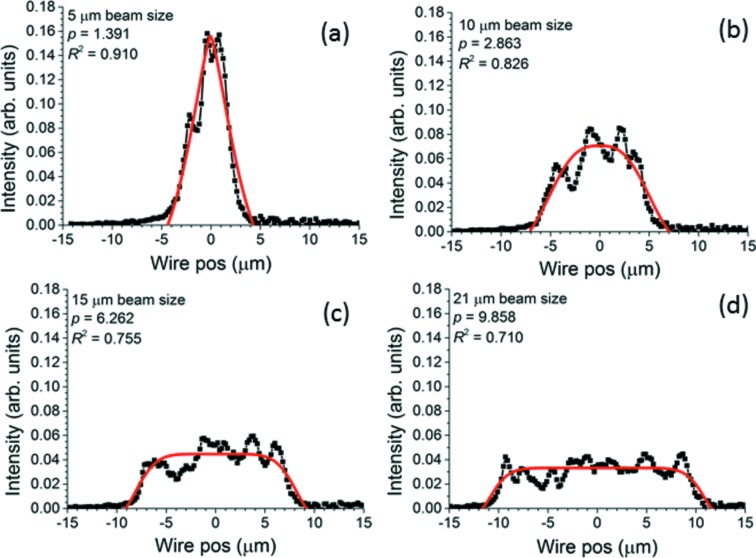
Simple concave modification: beam expansion to (*a*) 5 µm, (*b*) 10 µm, (*c*) 15 µm and (*d*) 21 µm at mirror’s focal point (approximately 0.4 m downstream from center of mirror). Black curves with dots are measured profiles. Thick solid red curves are best-fit super-Gaussians. Values of *p* and *R* ^2^ are shown in labels. Note the large beam sizes, but also the high striation level indicated by the low goodness of fit *R* ^2^.

Qualitatively, the experimental beam profile closest to a flat top with minimal striation is obtained using the seven-segment modification in Fig. 10(*d*) ▸ when the knife-edge is placed 385.6 mm downstream from the center of the mirror chamber. Fig. 11 ▸ contrasts it with both a typical profile obtained using the five-segment modification and a beam profile measured when a surface modification of uniform curvature was applied to the EEM bimorph mirror. Because the curvature no longer needs to change sign at the electrode junctions, the beam can be enlarged far more than with the re-entrant surface modification without unacceptably large voltage differentials between adjacent electrodes. Examples are shown in Fig. 12 ▸. However, even at a beam size of 5 µm, only slightly larger than that achieved using the re-entrant modifications, the beam at the nominal focus created by the modification of uniform curvature clearly shows three peaks and is not a good match to the relatively flat super-Gaussian obtained by fitting the wire scan for the best seven-segment modification, as seen by comparing the *R* ^2^ values (Fig. 11*c* ▸). At larger beam sizes the striations are even more visible. The best profiles achieved with the seven-segment modification are smoother. Moreover, unlike the five-segment modification, which produces mainly hot edges and little other structure in the reflected beam at the focus, the modification of uniform curvature produces striations throughout the beam. Finally, the hot edges produced by a re-entrant modification are easily seen to arise from the rounding of the curvature close to the breakpoints of that modification. They will therefore be similar for every mirror (assuming that the polishing errors are not larger than the modification). The striations produced by a modification of uniform curvature, however, do not arise from the modification itself, but from the mid- to long-wavelength polishing errors, which are uncontrolled and different for every mirror.

**Figure 13:**
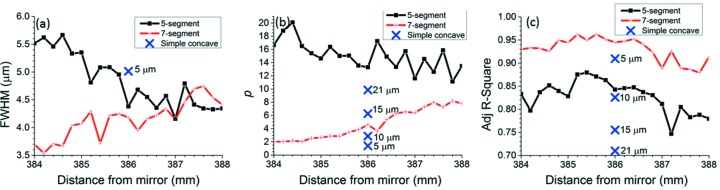
Figures of merit determined from the best-fit super-Gaussians of experimental beam profiles. (*a*) FWHM is 4–5 um as required, (*b*) *p* (higher value shows a flatter top super-Gaussian), (*c*) *R* ^2^ (closer to 1 indicates lower striation). As expected, the seven-segment modification provides a better approximation to a flat-top intensity profile. The crosses show the results for the earlier set of knife-edge scans displayed in Fig. 12 ▸. The label for each cross shows the beam size. The placement of the crosses at 386 mm on the graphs is only to allow them to be compared with the results for the re-entrant modifications.

An examination of the figures of merit determined by the super-Gaussian fits will place the observations above on a quantitative basis. Fig. 13 ▸ shows FWHM, *p* and *R* ^2^ for all sets of data, including the fits reported in Figs. 9 ▸–12 ▸. The FWHM in Fig. 13(*a*) ▸ confirms that the beam size in the center of the range of distances from the mirror is indeed 4–5 µm as required for all surface modifications applied to the mirror in this paper. The trend of the FWHM with increasing distance from the mirror (decreasing for the five-segment modification and increasing for the seven-segment modification) arises from the single long segment around the center of the mirror, which curves in opposite directions in the two modifications. The values of *p* and *R* ^2^ allow the surface modifications to be clearly ranked according to their ability to produce a flat-topped beam without striation. The uniform curvature modification for 5 µm beam yields the lowest value of *p* (*i.e.* the most triangular super-Gaussian) and the middle value of *R* ^2^ (*i.e.* medium striation). The five-segment modification yields the highest value of *p* (*i.e.* the most flat-topped super-Gaussian), but its *R* ^2^ is the lowest of all, evidently because of the strong hot edges it produces. The seven-segment modification produces profiles of medium *p*, but this is still better than the modification of uniform curvature, and Fig. 11(*a*) ▸ shows that the best-fit super-Gaussian for this case is still quite flat. Moreover, the seven-segment modification consistently produces the highest value of *R* ^2^ and hence the best fit to the super-Gaussian model. All considered, the uniform curvature modification performs least well and the seven-segment modification performs best. The best profile from the seven-segment modification scores well in both flatness (*p* = 3.463) and low striation (*R* ^2^ = 0.962).

### 4.4. Modeling of beam striations using geometrical optics   

**Figure 14:**
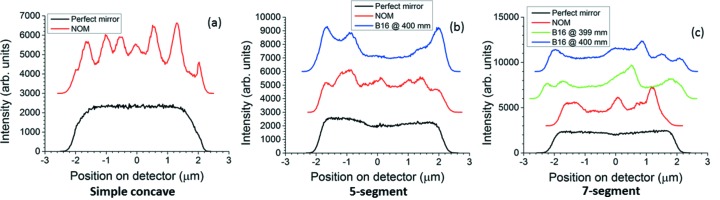
(*a*) Simple concave defocusing for a 5 µm beam size at focus (400 mm from center of mirror), (*b*) five-segment for 4 µm beam size at focus, (*c*) seven-segment for 4 µm beam size at focus. Beam profiles calculated by *SHADOW* (Sánchez del Río *et al.*, 2011 ▸). Black (bottom) curves represent perfect mirrors with no polishing errors. Slight non-uniformities are due to restriction of illumination over super-polished length, leaving the far edges of the re-entrant modifications dark. Subsequent curves in each plot are based on measurements of the mirror’s real surface (including polishing errors) using Diamond-NOM and X-ray speckle tracking on B16. For the B16 data, ray traces were performed with the detector at 399 mm and 400 mm from the center of the mirror.

Fig. 14 ▸ shows theoretical calculations of reflected beam profiles at or near the mirror focus (0.4 m downstream from mirror center) applying the geometrical optics program *SHADOW* (Sánchez del Río *et al.*, 2011 ▸) to various surface modifications on the mirror. The r.m.s. size (34.5 µm horizontal × 7.0 µm vertical) and emittance (2.6 nm rad horizontal × 0.008 nm rad vertical) of the electron beam source have been included. The theoretical profiles are similar to, but do not exactly match, the measured profiles for three reasons. First, the exact position of the mirror focus during the experiment was uncertain to within several millimeters because the precise center of the mirror was hidden inside the chamber, and Fig. 14(*c*) ▸ shows that the striations predicted for a measured re-entrant surface modification are strongly affected by even a 1 mm displacement of the detector off the focus. Second, the temperature and mounting conditions of the mirror on B16 are inevitably less well controlled than those on the Diamond-NOM, which is inside a stabilized cleanroom. The shape of the mirror could therefore have been distorted. Third, the X-ray speckle tracking technique used with the upstream diffuser conveniently provides the slope error of the mirror directly, but is known to be slightly less accurate than other versions of this technique which provide only curvature of the reflected wavefront. In addition, it should be mentioned that the optical components on the beamline may show some vibration introduced by their environment. If it were large enough, it would expand and smooth out the measured reflected beam profiles. However, the comparisons of this section indicate that the vibrations are not in fact influencing the profiles significantly.

Nevertheless, much about the experimental reflected beam profiles can be understood from the simulations. The number and strength of the spikes within each profile predicted by geometrical optics for the measured five-segment and seven-segment modifications are in accordance with those measured in the knife-edge scans, indicating that geometrical optics can at least qualitatively explain the measured beam profiles. Theoretical profiles in Fig. 14 ▸ do not reproduce the measured positions of the spikes very well, but geometrical optics even explains this discrepancy by providing a simple formula for the positions of the spikes: if *q* << *L*, *S*(*x*) is the measured slope modification, and *x*_c_ is a point on the mirror such that *S*′(*x* = *x*_c_) = 0, then a spike will appear in the reflected beam profile at a distance 2*qS*(*x*_c_) from the profile’s center, as shown by Nicolas & García (2013 ▸) and Sutter *et al.* (2014 ▸). This occurs because in the neighborhood of *x* = *x*_c_ the curvature is exactly that of the ideal elliptical arc for focusing on the detector, but the local slope is slightly changed from that of the ideal ellipse so that the rays from that part of the mirror are deflected to a focus at a slightly different point. Looking at the measured five-segment and seven-segment surface modifications, one would predict that the main creators of spikes are the areas of the mirror near the breakpoints, which are rounded (that is, have finite curvature throughout) and not sharp as are the desired ideal modifications. This explains the concentration of intensity at the edges of the profiles produced by the five-segment modification, where the rounding near the breakpoints is strong. It also explains why these ‘hot edges’ are so much less prominent in the profiles produced by the seven-segment modification: the change in curvature around the breakpoints is more abrupt and therefore closer to the ideal behavior.

The absence of other structure than the hot edges in the measured five-segment profiles can be explained by the absence of regions other than those near the breakpoints where the derivative of the measured slope modification in Fig. 8(*a*) ▸ becomes zero. It can also be explained by a fortuitous lining up of the slope modification’s maxima and minima. Only in Figs. 9(*d*) and 9(*e*) ▸ does a somewhat more complex structure, similar to the theoretical calculation in Fig. 14(*b*) ▸, start to appear. There, spikes produced by the slope minima no longer coincide on the detector, and neither do those produced by the slope maxima. The slope minima and maxima of the measured seven-segment modification do not line up so well, and so their spikes do not overlap. This no doubt is responsible for the more complex beam structures measured for this modification in Fig. 10 ▸. The asymmetric ‘sawtooth’ spikes in Fig. 10(*d*) ▸ match the theoretical example in Fig. 14(*c*) ▸ (second trace from top), which appears due to the partial overlap of a stronger spike with a somewhat weaker one. The strong central spike in Fig. 10(*e*) ▸ is certainly due to the flat region at the center of the measured seven-segment slope modification of Fig. 8(*b*) ▸, which here produces a focus at the detector. This lies at the junction between the joined electrodes 4 and 5 and is certainly an imprint effect. The largest discrepancy between theory and experiment is in the profile for the simple concave modification [compare Fig. 12(*a*) ▸ with Fig. 14(*a*) ▸]. This is probably caused by an uncertain distortion of the mirror’s surface due to the less well controlled environment at B16. However, the larger beam expansions in Figs. 12(*b*)–12(*d*) ▸ show well separated spikes much more like those calculated in Fig. 14 ▸(*a*).

**Figure 15:**
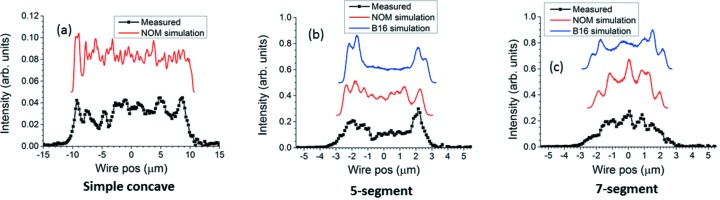
(*a*) Simple concave for 21 µm beam size at focus, (*b*) five-segment for 4 µm at 384.6 mm from center of mirror chamber, (*c*) seven-segment for 4 µm at 385.6 mm from mirror chamber: comparisons of measured beam profiles (bottom) with *SHADOW* simulations using measured slope modifications from NOM and (where available) X-ray speckle tracking at B16, displayed in each plot.

Fig. 15 ▸ compares three selected measured beam profiles generated by a simple concave, five-segment and seven-segment surface modification with theoretical profiles of the same width calculated using *SHADOW* on mirror figures measured by the Diamond-NOM and by X-ray speckle tracking at B16. Although striations within each plot do not precisely agree, they are of similar strength. For the simple concave modification, the 21 µm beam size was chosen on the assumption that any distortion of the mirror on B16 due to mounting and temperature would have been small and of gradual spatial variation, and hence could be neglected more easily for a large modification than for the small modification that produced the 5 µm beam. For the five-segment and seven-segment modifications, the measured plots selected were those that best matched simulations at focus in Fig. 14 ▸.

Spikes in the beam profile at the focus could in theory be introduced by locally flat regions of the mirror that are too short to be corrected by setting the voltages. However, neither the NOM data nor the X-ray speckle tracking data in Fig. 8 ▸ give convincing evidence that such regions actually exist. Furthermore, the beam profiles produced by the five-segment modification show little striation other than the hot edges. The profiles produced by the seven-segment modification are somewhat more difficult to evaluate, but the simulated profiles of Fig. 15(*c*) ▸ show six distinct peaks, one for each breakpoint. The measured profile shown in that figure does not have significantly more structure than the simulations.

As a final remark, it is clear that the observed X-ray beam striations are due to the polishing errors on the mirror, which continue to limit performance even when at the state-of-the-art nanometer level. Visible light laser beams present much lower levels of striation when shaped because their longer wavelengths make optical polishing errors less significant.

## 5. Conclusions   

This article expands on previous research into re-entrant surface modifications applied to a mirror for beam expansion with three innovations: the construction of re-entrant modifications on segments of any possible length and location, the deliberate choice of breakpoints to coincide with the position of the piezos and the polishing errors of a real deformable bimorph mirror, and the application of well suited re-entrant surfaces to such a mirror. Although the largest achievable beam size at the focal position is smaller with the re-entrant modification than with uniform curvature, the expanded beam profile has a better combination of flatness and lower striation, especially when a larger number of segments are used. In addition, striations that do appear when a re-entrant surface modification is used depend chiefly on the rounding of the curvature at the segment endpoints, rather than on the random polishing errors as is the case with a modification of uniform curvature. The re-entrant surface modifications therefore offer greater predictability when applied to different deformable bimorph mirrors. Finally, the new type of re-entrant surface modification permits the beam size at the focal position to be varied through a continuous range by applying appropriate permissible voltages to the bimorph mirror.

A few general statements may be made about the factors to consider in order to optimize the number of segments. For a chosen beam size at the focus, the maximum and minimum slope of the re-entrant modification is independent of the number of segments. However, the height of the re-entrant modification will decrease as the number of segments increases. A practical limit on the number of segments is therefore reached when the height of the re-entrant modification drops to a level comparable with the height variation of the polishing errors. Laundy *et al.* (2016 ▸) point out that a periodic surface modification will behave like a diffraction grating if its period is too short. A pattern of diffraction fringes would then appear in the beam profile and would vary depending on the selected photon energy. However, here too the possibility of applying non-periodic re-entrant surface modifications may be useful as they will break up the diffraction patterns.

The EEM bimorph mirror tested for this article is actually quite restricted in its range of possible beam sizes because its PRFs are considerably smaller than those of other deformable bimorph mirrors that have been measured at Diamond. Deformable bimorph mirrors with larger PRFs could expand the beam more. Some of these mirrors have PRFs that lack the strong overshoot and undershoot of the EEM PRFs around the region of the electrode; it is possible that they would experience less rounding of the curvature at the segment endpoints and hence be less likely to cause hot edges. The maximum achievable beam size at the focus would be limited by the magnitude of the PRFs, the maximum and minimum voltages allowed, and the maximum allowed difference between voltages on adjacent electrodes. The experience reported here with the EEM bimorph mirror shows clearly that the last factor becomes more and more restrictive as the number of segments is increased.

## References

[bb1] Alcock, S. G., Sawhney, K. J. S., Scott, S., Pedersen, U., Walton, R., Siewert, F., Zeschke, T., Senf, F., Noll, T. & Lammert, H. (2010). *Nucl. Instrum. Methods Phys. Res. A*, **616**, 224–228.

[bb2] Alcock, S. G., Sutter, J. P., Sawhney, K. J. S., Hall, D. R., McAuley, K. & Sorensen, T. (2013). *Nucl. Instrum. Methods Phys. Res. A*, **710**, 87–92.

[bb3] Bahrdt, J. (1997). *Appl. Opt.* **36**, 4367–4381. 10.1364/ao.36.004367 18259224

[bb4] Berujon, S., Wang, H., Alcock, S. & Sawhney, K. (2014). *Opt. Express*, **22**, 6438–6446. 10.1364/OE.22.006438 24663992

[bb5] Born, M. & Wolf, E. (1999). *Principles of Optics*, 7th ed. Cambridge University Press.

[bb6] Bowler, M. A. & Higgins, S. P. (2009). *FOCUS – a new wavefront propagation code. SMEXOS Conference Seminar*, ESRF, Grenoble, France, February 2009. (Available at http://www.esrf.eu/files/live/sites/www/files/events/conferences/2009/SMEXOS/talkBowler.pdf.)

[bb7] Chubar, O. & Elleaume, P. (1998). *Proceedings of Sixth European Particle Accelerator Conference (EPAC ’98)*, edited by S. Myers, L. Liljeby, C. Petit-Jean-Genaz, J. Poole and K.-G. Rensfeldt, pp. 1177–1179. Bristol: Institute of Physics.

[bb8] Hignette, O., Freund, A. & Chinchio, E. (1997). *Proc. SPIE*, **3152**, 188–199.

[bb9] Laundy, D., Alianelli, L., Sutter, J., Evans, G. & Sawhney, K. (2015). *Opt. Express*, **23**, 1576–1584. 10.1364/OE.23.001576 25835915

[bb10] Laundy, D., Sawhney, K., Nistea, I., Alcock, S. G., Pape, I., Sutter, J., Alianelli, L. & Evans, G. (2016). *Rev. Sci. Instrum.* **87**, 051802. 10.1063/1.4950732 27250369

[bb11] MathWorks (2004). *Matlab Version 7.0.1.24704 (R14) Service Pack 1.* MathWorks Inc., Massachusetts, USA.

[bb12] Nicolas, J. & García, G. (2013). *Proc. SPIE*, **8848**, 884810.

[bb13] OriginLab Corporation (2012). *Origin Pro 9.0.0.* OriginLab Corporation, Massachusetts, USA.

[bb14] Raimondi, L. & Spiga, D. (2015). *Astron. Astrophys.* **573**, A22.

[bb15] Sanchez del Rio, M., Canestrari, N., Jiang, F. & Cerrina, F. (2011). *J. Synchrotron Rad.* **18**, 708–716. 10.1107/S0909049511026306 PMC3267628 21862849

[bb16] Sawhney, K., Alcock, S., Sutter, J., Berujon, S., Wang, H. & Signorato, R. (2013). *J. Phys. Conf. Ser.* **425**, 052026.

[bb17] Sawhney, K. J. S., Dolbnya, I. P., Tiwari, M. K., Alianelli, L., Scott, S. M., Preece, G. M., Pedersen, U. K. & Walton, R. D. (2010). *AIP Conf. Proc.* **1234**, 387–390.

[bb18] Scheer, M. (2008). PhD thesis, Humboldt-Universität zu Berlin, Germany. (Available at http://edoc.hu-berlin.de/dissertationen/scheer-michael-2008–11-17/PDF/scheer.pdf.)

[bb19] Shealy, D. L. & Hoffnagle, J. A. (2005). *Proc. SPIE*, **5876**, 58760D.

[bb20] Signorato, R., Hignette, O. & Goulon, J. (1998). *J. Synchrotron Rad.* **5**, 797–800. 10.1107/S0909049597012843 15263657

[bb21] Signorato, R. & Ishikawa, T. (2001). *Nucl. Instrum. Methods Phys. Res. A*, **467**–**468**, 271–274.

[bb22] Spiga, D. & Raimondi, L. (2014). *Proc. SPIE*, **9209**, 92090E.

[bb23] Spiga, D., Raimondi, L., Svetina, C. & Zangrando, M. (2013). *Nucl. Instrum. Methods Phys. Res. A*, **710**, 125–130.

[bb24] Susini, J., Labergerie, D. & Zhang, L. (1995). *Rev. Sci. Instrum.* **66**, 2229–2231.

[bb25] Sutter, J. P., Alcock, S. G., Kashyap, Y., Nistea, I., Wang, H. & Sawhney, K. (2016). *Proc. SPIE*, **9965**. 99650E. 10.1107/S1600577516013308 PMC5082462 27787239

[bb26] Sutter, J. P., Alcock, S. G., Rust, F., Wang, H. & Sawhney, K. (2014). *Proc. SPIE*, **9208**, 92080G.

[bb27] Sutter, J. P., Alcock, S. G. & Sawhney, K. J. S. (2011). *Proc. SPIE*, **8139**, 813906.

[bb28] Sutter, J. P., Amboage, M., Hayama, S. & Díaz-Moreno, S. (2010). *Nucl. Instrum. Methods Phys. Res. A*, **621**, 627–636.

[bb29] Svetina, C., Sostero, G., Sergo, R., Borghes, R., Callegari, C., D’Amico, F., Bencivenga, F., Masciovecchio, C., Di Cicco, A. & Cocco, D. (2011). *Nucl. Instrum. Methods Phys. Res. A*, **635**, S12–S15.

[bb30] Wang, H., Kashyap, Y., Laundy, D. & Sawhney, K. (2015). *J. Synchrotron Rad.* **22**, 925–929. 10.1107/S1600577515006657 PMC4489535 26134795

[bb31] Wang, H., Sawhney, K., Berujon, S., Sutter, J., Alcock, S. G., Wagner, U. & Rau, C. (2014). *Opt. Lett.* **39**, 2518–2521. 10.1364/OL.39.002518 24979033

[bb32] Wang, H., Sutter, J. & Sawhney, K. (2015). *Opt. Express*, **23**, 1605–1614. 10.1364/OE.23.001605 25835918

[bb33] Yamauchi, K., Mimura, H., Inagaki, K. & Mori, Y. (2002). *Rev. Sci. Instrum.* **73**, 4028–4033.

